# Hyperopia-Correcting Phototherapeutic Keratectomy and Its Comparison With Conventional Phototherapeutic Keratectomy

**DOI:** 10.3389/fmed.2022.708188

**Published:** 2022-03-10

**Authors:** Hideki Hayakawa, Kazutaka Kamiya, Tatsuhiko Tsujisawa, Masahide Takahashi, Nobuyuki Shoji

**Affiliations:** ^1^Department of Ophthalmology, Kitasato University, School of Medicine, Sagamihara, Japan; ^2^Visual Physiology, Kitasato University, School of Allied Health Sciences, Sagamihara, Japan

**Keywords:** HC-PTK, conventional PTK, hyperopia, granular corneal dystrophy, band-shaped keratopathy

## Abstract

**Purpose:**

To evaluate hyperopia-correcting phototherapeutic keratectomy (HC-PTK) and to compare the visual and refractive outcomes of HC-PTK and conventional PTK.

**Methods:**

This study comprised a total of 72 eyes of 72 consecutive patients who underwent HC-PTK and conventional PTK for granular corneal dystrophy or band-shaped keratopathy. Preoperatively and 6 months postoperatively, we assessed visual acuity, manifest refraction, and mean keratometry, as well as postoperative corneal higher-order aberrations and adverse events in each PTK group, and compared these metrics between the two groups.

**Results:**

LogMAR BSCVA significantly improved from 0.43 ± 0.47 preoperatively to 0.21 ± 0.38 postoperatively in the HC-PTK group (Wilcoxon signed-rank test, *p* < 0.001). It was also significantly improved from 0.22 ± 0.21 preoperatively to 0.15 ± 0.12 postoperatively in the conventional PTK group (*p* = 0.031). Mean refraction significantly changed from 0.27 ± 1.55 diopter (D) preoperatively to 0.50 ± 1.77 D postoperatively, in the HC-PTK group (*p* = 0.313). By contrast, it was significantly hyperopic from −0.15 ± 2.41 D preoperatively to 1.45 ± 2.46 D postoperatively, in the conventional PTK group (*p* < 0.001). No significant complications occurred in any case during the follow-up period.

**Conclusion:**

Both HC-PTK and conventional PTK showed a significant improvement of BSCVA and no vision-threatening complications at any time in this series. HC-PTK significantly reduced a hyperopic shift in refraction compared with conventional PTK, suggesting its viability for patients requiring PTK, especially in consideration of preventing this hyperopic issue.

## Background

Phototherapeutic keratectomy (PTK) has been widely acknowledged to be a safe and effective means to remove diseased tissue by the use of excimer laser photoablation in eyes having opaque corneas such as granular corneal dystrophy (GCD) and band-shaped keratopathy (BSK) (1–7). A hyperopic shift in refraction can occur after PTK surgery due to the flattening of the cornea by laser photoablation, although its shift largely depends on the excimer laser system (1, 6–10). Accordingly, it is one of the ongoing concerns that have to be taken into consideration, especially in eyes undergoing cataract surgery and in eyes requiring unilateral PTK. Therefore, it is clinically helpful to prevent this hyperopic shift in such patients, in order to maximize unaided vision and patient satisfaction. The present study aims to prospectively compare the clinical outcomes of our devised hyperopia-correcting PTK (HC-PTK) and conventional PTK for GCD and BSK, with special attention to refractive changes.

## Materials and Methods

### Study Population

The study protocol was registered with the University Hospital Medical Information Network Clinical Trial Registry (000044107). This prospective observational study comprised a total of 72 eyes of 72 consecutive patients, who underwent HC-PTK (38 eyes) or conventional PTK (34 eyes) for the treatment of GCD or BSK at Kitasato University Hospital, and who completed at least a 6-month follow-up. The estimated total postoperative corneal thickness was at least 400 μm or more to prevent the occurrence of iatrogenic keratectasia. Eyes with any history of ocular surgery, ocular trauma, or other concomitant eye diseases, except for inactive uveitis, mild glaucoma, or macular atrophy for BSK, which could affect the measurements of refraction, were excluded from the study. Eyes with keratoconus were also excluded from the study by using the keratoconus screening test of Placido disk videokeratography (TMS-4, Tomey Co., Inc., Aichi, Japan). The sample size in this study offered 84.7% statistical power at the 5% level to detect a 1-diopter (D) difference in refraction, while the standard deviation of the mean difference was 1.4 D. This prospective observational study was approved by the Institutional Review Board at Kitasato University (B18-223) and followed the tenets of the Declaration of Helsinki. Written informed consent for this treatment was obtained from all patients after an explanation of the nature and possible consequences.

### Surgical Procedures

For conventional PTK, we conducted it with the VISX Star S4 excimer laser system (Johnson & Johnson Vision, Santa Ana, CA, United States) by one experienced surgeon (KK), by using the following parameters: wavelength, 193 nm; fluency, 160 mJ/cm^2^; repetition rate, 10 Hz; ablation zone diameter, and 6.5 mm (including transition zone, 0.5 mm) using the photorefractive keratectomy (PRK) mode, as described previously (11, 12). The intended ablation depth was individually determined based on the depth of pathology using an anterior segment optical tomographer (CASIA2, Tomey Co., Inc., Aichi, Japan), and the corrected diopter was inversely calculated with the ablation depth, when the estimated corneal thickness was 450 μm or more. We used the transepithelial technique for the removal of the corneal epithelium (in a depth of 50 μm).

**FIGURE 1:**
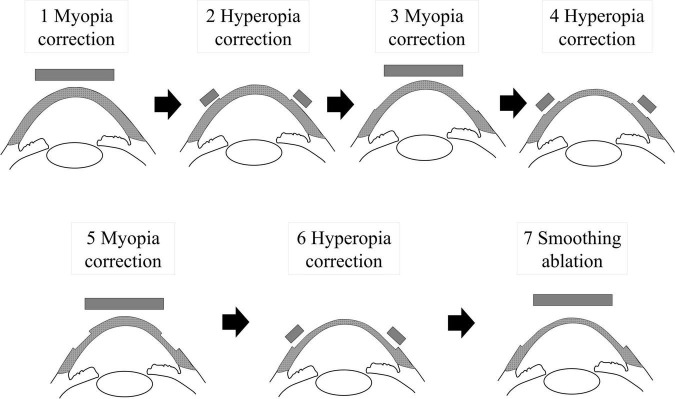
A schematic diagram of hyperopia-correcting phototherapeutic keratectomy (HC-PTK).

For HC-PTK, we employed the same settings of the excimer laser by the same experienced surgeon. We alternatively performed photorefractive keratectomy for myopia (each −1.25 to −1.5 D, diameter 6.5 mm) and hyperopia (each +1 D, diameter 6.0–9.0 mm) three times (step 1–6), followed by PTK treatment with a depth of 20 μm (diameter 6.5 mm) to smoothen the ocular surface (step 7), without the use of the transepithelial technique (Figure 1). This ablation profile was primarily devised by *in vitro* preliminary experiments of the morphological analysis of a flat glass plate by using this excimer laser system, in order to effectively remove the tissue with the least induction of the dioptric change after surgical ablation.

After both PTK surgery techniques, steroidal (0.1% fluorometholone, Flumethasone™, Santen, Osaka, Japan) and antibiotic (1.5% levofloxacin, Cravit™, Santen) medications were topically administered four times daily for 1 week, after insertion of a soft contact lens, and the dose was steadily reduced thereafter.

### Outcome Measures

Preoperatively and 6 months postoperatively, we assessed the logarithm of the minimum angle of resolution (logMAR) uncorrected visual acuity (UCVA), and logMAR best spectacle-corrected visual acuity (BSCVA), manifest refraction (spherical equivalent), mean keratometry, and central corneal thickness, in each PTK group. We also evaluated corneal higher-order aberrations (HOAs) for a 4-mm pupil postoperatively using the Scheimpflug camera system (Pentacam HR, Oculus, Germany), when we could obtain reliable and reproducible measurements. For subgroup analysis, we also determined the above parameters at the final follow-up (spanning more than 6 months) in the HC-PTK group.

### Statistical Analysis

We conducted statistical analyses by using a statistical software (Bell Curve for Excel, Social Survey Research Information Co., Ltd., Tokyo, Japan). We first checked the normal distribution of the data by the Shapiro–Wilk test. Since we confirmed that the data were not normally distributed in this population, we applied the Wilcoxon-signed rank test to compare the pre- and post-surgical data, and the Mann-Whitney U-test to compare the data between the two groups. We also applied the Spearman’s rank correlation test to assess the relationships between the two variables. The results are expressed as mean ± standard deviation, and a value of *p* < 0.05 was deemed statistically significant.

## Results

### Study Population

Table 1 shows the preoperative demographics of the study population. The patient age at the time of surgery was 73.5 ± 9.6 and 73.9 ± 8.9 years in the HC-PTK and conventional PTK groups, respectively. All surgeries were uneventful, and no intraoperative complications were observed. We found no significant differences in the preoperative demographics between the two groups. Table 2 also shows the postoperative demographics of the study population. The total depth was 34.7 ± 5.7 μm in the HC-PTK group, and 37.2 ± 5.8 μm in the conventional PTK group (*p* = 0.298).

**TABLE 1:** Preoperative demographics of the study population in eyes with undergoing hyperopia-correcting phototherapeutic keratectomy (HC-PTK) and conventional phototherapeutic keratectomy (PTK).

Preoperative	HC-PTK	Conventional PTK	*P*-value*
Age	73.5 ± 9.6 years	73.9 ± 8.9 years	0.993
Gender (Male: Female)	7: 16	7: 20	0.824
LogMAR UCVA	0.63 ± 0.43	0.62 ± 0.38	0.780
LogMAR BSCVA	0.43 ± 0.47	0.22 ± 0.21	0.115
Manifest spherical equivalent	0.27 ± 1.55 D	−0.15 ± 2.41 D	0.835
Manifest cylinder	−0.80 ± 0.98 D	−0.67 ± 0.66 D	0.995
Corneal refractive power	43.8 ± 1.41 D	44.3 ± 1.41 D	0.143
Corneal higher-order aberrations	0.64 ± 0.29 μm	0.56 ± 0.37 μm	0.101
Central corneal thickness	531.9 ± 38.9 μm	543. 0 ± 26.4 μm	0.256
Disease	GCD 13 eyes, BSK 25 eyes	GCD 12 eyes, BSK 22 eyes	0.885

LogMAR, logarithm of the minimum angle of resolution; UCVA, uncorrected visual acuity; BSCVA, best spectacle-corrected visual acuity; D, diopter; GCD, granular corneal dystrophy; BSK, band-shaped keratopathy. *Mann–Whitney U test.

**TABLE 2:** Postoperative demographics of the study population in eyes with undergoing hyperopia-correcting phototherapeutic keratectomy (HC-PTK) and conventional phototherapeutic keratectomy (PTK).

Postoperative	HC-PTK	Conventional PTK	*P*-value*
LogMAR UCVA	0.50 ± 0.39	0.68 ± 0.36	0.020
LogMAR BSCVA	0.21 ± 0.38	0.15 ± 0.12	0.228
Manifest spherical equivalent	0.50 ± 1.77 D	1.45 ± 2.46 D	0.040
Manifest cylinder	−0.80 ± 0.98 D	−0.70 ± 0.72 D	0.901
Corneal refractive power	43.0 ± 1.8 D	42.1 ± 1.8 D	0.144
Corneal higher-order aberrations	0.60 ± 0.39 μm	0.46 ± 0.24 μm	0.250
Central corneal thickness	491.9 ± 36.3 μm	494.1 ± 37.7 μm	1.000

LogMAR, logarithm of the minimum angle of resolution; UCVA, uncorrected visual acuity; BSCVA, best spectacle-corrected visual acuity; D, diopter. *Mann–Whitney U test.

### Visual Outcomes

**FIGURE 2:**
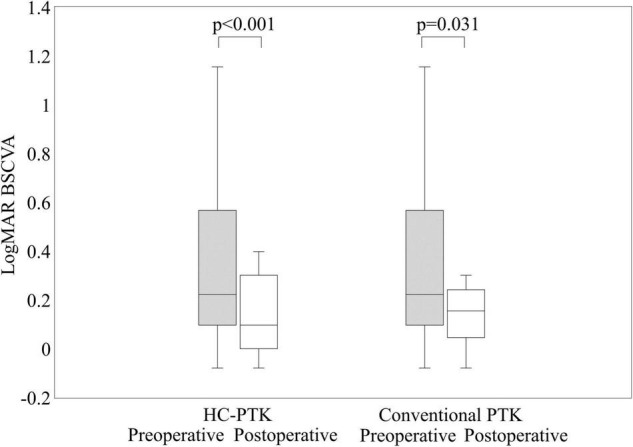
Preoperative and postoperative visual outcomes in eyes undergoing hyperopia-correcting phototherapeutic keratectomy (HC-PTK) and conventional phototherapeutic keratectomy (PTK).

Figure 2 shows the preoperative and postoperative logMAR BSCVA in the HC-PTK and conventional PTK groups. LogMAR UCVA improved, but not significantly, from 0.63 ± 0.43 preoperatively, to 0.50 ± 0.39 postoperatively in the HC-PTK group (Wilcoxon signed-rank test, *p* = 0.119). It worsened, but not significantly, from 0.62 ± 0.38 preoperatively, to 0.68 ± 0.36 postoperatively in the conventional PTK group (*p* = 0.399). We found no significant differences in the change of UCVA between the two groups (*p* = 0.171). LogMAR BSCVA significantly improved from 0.43 ± 0.47 preoperatively, to 0.21 ± 0.38 postoperatively in the HC-PTK group (*p* < 0.001). It also significantly improved from 0.22 ± 0.21 preoperatively to 0.15 ± 0.12 postoperatively in the conventional PTK group (*p* = 0.031). We also found no significant differences in the improvement of BSCVA between the two groups (*p* = 0.090).

### Refractive Outcomes

**FIGURE 3:**
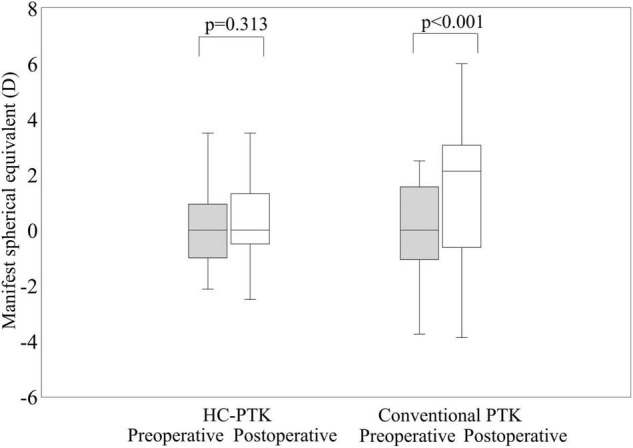
Preoperative and postoperative refractive outcomes in eyes undergoing hyperopia-correcting phototherapeutic keratectomy (HC-PTK) and conventional phototherapeutic keratectomy (PTK).

Figure 3 shows the preoperative and postoperative manifest spherical equivalent refraction in the HC-PTK and conventional PTK groups. Manifest spherical equivalent did not significantly change from 0.27 ± 1.55 D preoperatively to 0.50 ± 1.77 D postoperatively, in the HC-PTK group (*p* = 0.313). By contrast, it became significantly hyperopic from −0.15 ± 2.41 D preoperatively to 1.45 ± 2.46 D postoperatively, in the conventional PTK group (*p* < 0.001).

### Corneal Refractive Power and Higher-Order Aberrations

**FIGURE 4:**
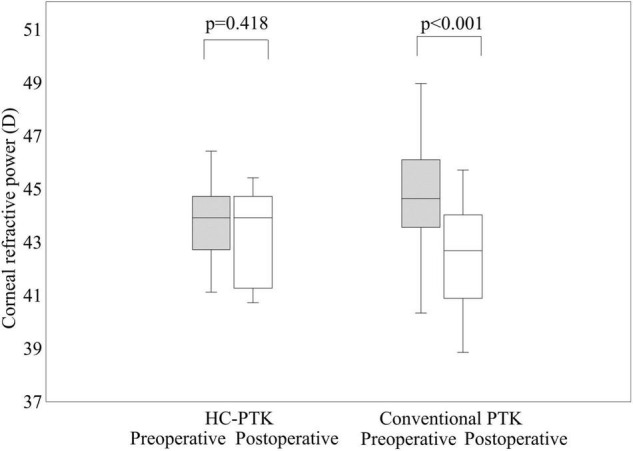
Preoperative and postoperative corneal refractive power in eyes undergoing hyperopia-correcting phototherapeutic keratectomy (HC-PTK) and conventional phototherapeutic keratectomy (PTK).

The mean keratometric readings did not significantly change from 43.8 ± 1.4 D preoperatively to 43.0 ± 1.8 D postoperatively, in the HC-PTK group (*p* = 0.418). In contrast, they significantly decreased from 44.3 ± 1.4 D preoperatively to 42.1 ± 1.8 D postoperatively, in the conventional PTK group (*p* < 0.001) (Figure 4).

We found no significant change in corneal HOAs in the HC-PTK group (*p* = 0.248) or the conventional PTK group (*p* = 0.160). We also found no significant correlations between surgically induced HOAs and the change in BSCVA in the HC-PTK group (*r* = −0.213, *p* = 0.576) or in the conventional PTK group (*r* = 0.017, *p* = 0.923).

### Subgroup Analysis

Table 3 shows the final follow-up (spanning more than 6 months) demographic data of 19 eyes (50%) in the HC-PTK group. The observation period was 8.4 ± 2.7 months. We found similar visual and refractive outcomes at 6 months and at the final follow-up (*p* = 0.836 for UCVA, *p* = 0.591 for BSCVA, *p* = 0.971 for manifest equivalent, *p* = 0.921 for manifest cylinder, *p* = 0.637 for mean keratometry, *p* = 0.920 for corneal HOAs, and *p* = 0.637 for central corneal thickness).

**TABLE 3:** Postoperative demographics of the subgroup population in eyes with undergoing hyperopia-correcting phototherapeutic keratectomy (HC-PTK) and conventional phototherapeutic keratectomy (PTK) at the final follow-up (spanning more than 6 months).

Final follow-up	HC-PTK
LogMAR UCVA	0.44 ± 0.36
LogMAR BSCVA	0.19 ± 0.31
Manifest spherical equivalent	0.25 ± 1.37 D
Manifest cylinder	−0.74 ± 0.82 D
Corneal refractive power	44.0 ± 0.2 D
Corneal higher-order aberrations	0.76 ± 0.63 μm
Central corneal thickness	479.0 ± 17.3 μm

*LogMAR, logarithm of the minimum angle of resolution; UCVA, uncorrected visual acuity; BSCVA, best spectacle-corrected visual acuity; D, diopter.*

### Adverse Events

We found transient haze formation (Fantes scale Grade 1) (13) in 5 eyes (13%) and 3 eyes (9%) 1 month postoperatively, in the HC-PTK and conventional PTK groups, respectively, but all these eyes recovered with time by the continued use of steroidal eye drops. Otherwise, we found no vision-threatening complications such as keratectasia, central island formation, or infectious keratitis, during the 6-month observation period.

## Discussion

In the present study, our results demonstrated that both HC-PTK and conventional PTK provided a significant improvement of BSCVA and that there were no significant intraoperative or postoperative complications, suggesting its viability as a surgical treatment for GCD and BSK. Interestingly, our results also showed that conventional PTK induced a significant hyperopic change, but that HC-PTK did not induce a significant change in refraction in such diseased patients. As far as we can ascertain, this is the first study to assess the clinical outcomes of HC-PTK, as well as to compare their outcomes with those of conventional PTK treatment. We believe that this information is clinically meaningful for improving unaided vision and subsequent patient satisfaction in post-PTK patients, since it is still challenging to prevent this hyperopic shift after PTK surgery in daily practice. We assume that this novel approach will be beneficial especially in eyes having IOL implantation or eyes requiring unilateral PTK, since the hyperopic shift can be problematic after PTK surgery, even in diseased patients having GCD and BSK.

There have so far been many published studies on the safety and the efficacy outcomes of PTK surgery, but, as summarized in Table 4, the number of studies reporting the refractive outcomes of PTK is still rather limited. Although the degree of a hyperopic shift after PTK can be influenced by various factors, such as the type of excimer laser system, the amount of the photoablation, and the type of the disease, it has been reported that conventional PTK usually induced a hyperopic shift in refraction by approximately 1.0–4.0 D, (1, 6–10) which is comparable with our current findings of conventional PTK. There have been some pilot studies to prevent a hyperopic shift after PTK surgery. Amano et al. (14) firstly demonstrated that PTK followed by hyperopic PRK was effective for the prevention of the postoperative hyperopic shift in refraction, but that the postoperative BSCVA after this technique was similar to that after conventional PTK. Li et al. (15) stated that a flying-spot excimer laser can perform PTK by combining myopic ablation and hyperopic astigmatism ablation and that knowledge of refractive correction larger than the nominal settings can prevent undesired large hyperopic outcomes. Nakamura et al. (16) showed that a sequential procedure composed of PTK, followed by smoothing ablation for reducing the corneal surface irregularities and PRK ablation for correcting refractive errors, was beneficial to prevent this hyperopic change in refraction, although they applied this surgical technique to only eyes having GCD. Interestingly, Tobalem et al. (17) recently reported that the Wavelight flying spot excimer laser system produces myopic outcomes following PTK and that both ablation depth and optical zone were independent variables with significant effects on the final visual outcome. We should be aware that the hyperopic shift in refraction mainly depends on the individual excimer laser system. The flying spot technology may have advantages over the broad beam technology in terms of preventing this hyperopic change after PTK. Our approach may be unique in that we alternatively applied a small amount of PRK ablation for myopia and hyperopia 3 times, to efficiently remove the diseased tissue, as well as to reduce the irregularity of the cornea as much as possible, and that we employed the PRK mode, instead of the PTK mode, (11) since the VISX Star S4 excimer laser system induced central island formation in as many as 56% of eyes even 1 year postoperatively, which significantly affects the postoperative visual recovery, when using the PTK mode (12).

**TABLE 4:** Summary of previous studies on the refractive outcomes of phototherapeutic keratectomy (PTK).

References	Mean age	Eyes	Excimer laser	Manufacture	Disease	Mean Hyperopic shift
Gartry et al. (1)	54	25	UV200	Summit Technology	Anterior corneal disease	2.85 D
Maloney et al. (6)	62	232	ExciMed OmniMed	Summit Technology	Band-shaped keratopathy, Salzmann’s nodular degeneration, keratoconus, epithelial basement membrane dystrophy, other dystrophies, corneal scar	1.25 ± 2.50 D
Starr et al. (7)	N.A.	45	VISX	VISX	Postinflammatory and postsurgical scars, stromal dystrophies, surface degeneration, pterygium, epithelial basement membrane dystrophy	2.81 D
Amm et al. (8)	N.A.	45	MEL 60	Aesculap Meditex	Recurrent corneal erosion, central corneal scars, corneal dystrophy, surface irregularities	1.70 D
Amano et al. (9)	64	31	VISX20/20	VISX	Band-shaped keratopathy, granular corneal dystrophy, corneal scar	0.28 ± 5.22 D
Dogru et al. (10)	68	112	EC-5000	Nidek	Avellino dystrophy, granular corneal dystrophy, lattice dystrophy, band-shaped keratopathy, corneal leucoma	3.42 ± 1.15 D
Amano et al. (14)	69.8	63	EC-5000	Nidek	Granular corneal dystrophy, band-shaped keratopathy	0.09 ± 1.23 D
Li et al. (15)	55.7	26	Wavelight Allegretto	Alcon	Post-refractive surgery complications, post-microbial keratitis scar, other types of scars, post-penetrating keratoplasty, granular corneal dystrophy, irregular astigmatism post-cataract surgery	0.07 ± 0.69 D
Nakamura et al. (16)	55.7	23	MEL80	Carl Zeiss Meditec	Granular corneal dystrophy	0.30 ± 0.99 D
Tobalem et al. (17)		58	Wavelight Allegretto	Alcon	Recurrent corneal erosion syndrome without dystrophy and Cogan corneal dystrophies	−1.052 ± 1.260 D
Current	73.5	38	Star S4	VISX	Granular corneal dystrophy, band-shaped keratopathy	0.23 ± 1.23 D

*N.A., not applicable; D, diopter.*

A topical ethylene-diamine-tetra-acetic acid (EDTA) chelation has been commonly used for eyes having BSK, as a cost-effective treatment, since it does not require an excimer laser system. Although this treatment did not induce a significant change in refraction, Al-Hity showed no evidence of a significant difference between the initial and final visual acuity following EDTA treatment (18). We assume that PTK has advantages over EDTA chelation in terms of visual recovery, since the former treatment can provide a smoother corneal surface than the latter treatment.

There are at least two limitations to this study. Firstly, the sample size was relatively small, since the number of the patients having GCD and BSK without concomitant eye diseases was still limited in a clinical setting, although the statistical power was 84.7% at the 5% level in the current study. Secondly, the preoperative background factors were not completely matched between the two groups, since this study was performed in a non-randomized observational fashion. A randomized controlled clinical trial may be necessary for further strengthening the authenticity of our results. Thirdly, it is still challenging to precisely obtain the preoperative refraction in such opaque eyes having advanced GCD or BSK in daily practice. However, experienced optometrists confirmed the reliability and reproducibility of the preoperative measurements in all patients. Fourthly, HC-PTK cannot adjust the ablation depth during surgery by observing the remaining corneal opacity, since the surgical technique needs to balance the rate of myopic and hyperopic correction.

## Conclusion

In summary, our results may support the view that both HC-PTK and conventional PTK showed a significant improvement of BSCVA, and HC-PTK significantly reduced a hyperopic shift in refraction compared with conventional PTK. These findings indicate that HC-PTK, instead of conventional PTK, may hold a promise for reducing the hyperopic shift in refraction in post-PTK patients. A further study with a large number of post-HC-PTK patients is necessary to confirm our findings.

## Data Availability Statement

The original contributions presented in the study are included in the article/Supplementary Material, further inquiries can be directed to the corresponding author/s.

## Ethics Statement

This prospective observational study was approved by the Institutional Review Board at Kitasato University (B18-223). The patients/participants provided their written informed consent to participate in this study.

## Author Contributions

KK and NS were involved in the design and conduct of the study. HH, TT, and MT were involved in collection, management, analysis, and interpretation of data. HH, KK, TT, MT, and NS were involved in preparation, review, and final approval of the manuscript. All authors contributed to the article and approved the submitted version.

## Conflict of Interest

The authors declare that the research was conducted in the absence of any commercial or financial relationships that could be construed as a potential conflict of interest.

## Publisher’s Note

All claims expressed in this article are solely those of the authors and do not necessarily represent those of their affiliated organizations, or those of the publisher, the editors and the reviewers. Any product that may be evaluated in this article, or claim that may be made by its manufacturer, is not guaranteed or endorsed by the publisher.
