# Supplementary material for: Hyperopia-Correcting Phototherapeutic Keratectomy and Its Comparison With Conventional Phototherapeutic Keratectomy
Source: Front Med (Lausanne). 2022 Mar 10;9:708188. doi: 10.3389/fmed.2022.708188 (PMC8960048; doi:10.3389/fmed.2022.708188)
Supplement: Supplementary file 1 [file Data_Sheet_1.PDF]

Surgery	Protocol	HOAs (6 mm)<br>(RMS)
Conventional PTK	① Epithelial removal 50 μm<br>② PRK φ 6.5 mm S-3.0D<br>Total depth 89 μm	4.66 μm
HC-PTK<br>Pattern A	PRK φ 6.5 mm<br>① S-1.5 D→②S+1.0 D→<br>③S-1.25 D④→⑤S+1.0 D→⑥S-1.25 D<br>⑦Smoothing 20 μm<br>Total depth 84 μm	3.72 μm
HC-PTK<br>Pattern B	PRK φ 6.5 mm<br>① S-4.0 D→②S+3.0 D<br>③Smoothing 20 μm<br>Total depth 81 μm	4.32 μm
